# Facial palsy as a manifestation of COVID‐19: A systematic review of cases

**DOI:** 10.1002/hsr2.887

**Published:** 2022-10-28

**Authors:** Aiman Khurshid, Maman Khurshid, Aruba Sohail, Imran Mansoor Raza, Muhammad Khubab Ahsan, Mir Umer Farooq Alam Shah, Anab Rehan Taseer, Abdulqadir J. Nashwan, Irfan Ullah

**Affiliations:** ^1^ Department of Forensic Medicine Abbasi Shaheed Hospital Karachi Pakistan; ^2^ Department of Internal Medicine Dow University of Health Sciences Karachi Pakistan; ^3^ Department of Internal Medicine Jinnah Medical and Dental College Karachi Pakistan; ^4^ Department of Psychiatric Medicine Dr Ruth KM Pfau Civil Hospital Karachi Pakistan; ^5^ Department of Pulmonology, Lady Reading Hospital (LRH) Peshawar Pakistan; ^6^ Hamad Medical Corporation Doha Qatar; ^7^ Kabir Medical College Gandhara University Peshawar Pakistan

**Keywords:** COVID‐19, facial palsy, neurological symptoms, SARS‐CoV‐2, systematic review

## Abstract

**Background and Aims:**

Facial palsy is a rare complication of the COVID‐19 infection. Herein, we conducted a systematic review of all published cases of facial palsy post‐COVID‐19 infection in an attempt to educate the general population and medical practitioners regarding the likely occurrence of facial palsy in COVID‐19 patients, its detection, effective treatment plan, and prognosis of the condition.

**Methods:**

We searched PubMed, Google Scholar, and Directory of Open Access Journals (DOAJ) from December 1, 2019 to September 21, 2021.

**Results:**

We included 49 studies bearing accounts of 75 cases who had facial palsy. The mean age of patients was 42.9 ± 19.59 years, with a male‐to‐female ratio of 8:7. The majority of the cases were reported from Brazil (*n* = 14), USA (*n* = 9), Turkey (*n* = 9), and Spain (*n* = 9). Noticeably, 30.14% of COVID‐19 patients were diagnosed with Guillain‐Barré syndrome. In total, 22.97% of patients complained of bilateral facial paralysis (*n* = 17), whereas ipsilateral paralysis was observed in 77.03% (*n* = 57). These were common complaints of Lagophthalmos, otalgia, facial drooping, dysarthria, and compromised forehead wrinkling. The treatment regimen mainly included the use of corticosteroids (*n* = 51) (69.86%), antivirals (*n* = 23) (31.51%), IVIG (*n* = 18) (24.66%), antibiotics (*n* = 13) (17.81%), antiretroviral (*n* = 9) (12.33%), and antimalarial (*n* = 8) (10.96%) medications. In all, 35.62% of patients (*n* = 26) adhered to a combination of antiviral and corticosteroid‐based therapy. Positive treatment outcomes were observed in 83.58% (*n* = 56) of cases. In contrast, 10 patients (14.93%) showed nonsignificant recovery, out of which 3 (4.48%) died from the disease.

**Conclusion:**

The association of facial palsy with COVID‐19 is controversial and therefore requires further investigation and published work to confirm a causal relationship. However, physicians should not overlook the likelihood of facial palsy post‐COVID‐19 infection and treat it accordingly.

## 1 INTRODUCTION

On December 31, 2019, a novel coronavirus was first identified in Wuhan, China, after reports of multiple cases of pneumonia among its people.^1^ This was the start of an outbreak that took the shape of a pandemic over a few months, owing to its rapid transmission through respiratory droplets. As of May 10, 2022, 6.53% of the world population (*n* = 515,748,861) has confirmed infection with COVID‐19, while 1.21% of these have lost their lives to the complications of COVID‐19.^2^

COVID‐19 patients commonly complain of fever, fatigue, nasal congestion, myalgia, anosmia, dry cough, ageusia, hemoptysis, dyspnea, and so forth.^3^ Under more serious circumstances, COVID‐19 can result in severe pneumonia, acute respiratory distress syndrome, sepsis, septic shock, multiple organ failure, and so forth.^4^ While these are some of the most widely reported complications of COVID‐19 infection, other less common ones have also surfaced, for example, hemophagocytic lymphohistiocytosis (HLH), vasculitis, central retinal vein occlusion, and so forth.^5^, ^6^, ^7^ Similarly, facial palsy has emerged as an unusual yet interesting complication of COVID‐19, whose pathophysiology is yet to be known.

Numerous case reports and series documenting facial palsy as a complication of COVID‐19 have been published. In addition, some systematic reviews have discussed the association of facial palsy with COVID‐19. However, none of these reviews collectively assessed all the cases of facial palsy secondary to COVID‐19. For instance, Gupta et al.^8^ included only those cases in which facial palsy was an isolated neurological finding. Therefore, in our systematic review, we aim to develop a stronger evidence base by including all the cases of facial palsy secondary to COVID‐19 that have been published to date. Moreover, we generated patient‐level data by including case reports to thoroughly evaluate the patient characteristics and clinical course of facial palsy secondary to COVID‐19. This will not only bridge the gap in literature but will also aid physicians in reaching a timely diagnosis and in devising treatment regimens that cater to the patients' individual needs.

## 2 METHODOLOGY

### 2.1 Literature review

Our work aligns with the Preferred Reporting Items for Systematic Reviews and Meta‐Analysis (PRISMA) checklist (Supporting Information: File S1).^9^ We searched PubMed, Google Scholar, and Directory of Open Access Journals (DOAJ) from December 1, 2019 to September 21, 2021 for published accounts of cases of facial palsy as a symptom of COVID‐19. Search terms were combined using appropriate Boolean operators. Our search strategy included keywords/subject headings pertaining to COVID‐19 (e.g., SARS‐CoV‐2 OR Coronavirus Disease 2019 OR COVID‐19 OR severe acute respiratory syndrome coronavirus 2 OR coronavirus infection) and facial palsy (e.g., facial palsy OR facial weakness OR facial paresis OR bell's palsy). The Reference section of included studies was also checked for completeness' sake. Please refer to our Supporting Information: File S1 for a detailed search strategy. Furthermore, this study is registered in the International prospective register of systematic reviews (PROSPERO) and holds the unique identifying number (UIN); CRD42022324693.^10^

### 2.2 Inclusion and exclusion criteria

Our search criteria included all case reports, case series, editorials, correspondence, and retrospective cohorts on the topic of facial palsy following COVID‐19 infection. Only work published in English and containing comprehensive detail of clinical presentation and progression of the condition in each patient was included in our systematic review. Studies bearing aggregate level data, language barriers, and incomplete detail of the condition were excluded from our study. The title, abstract, and full‐text screening were completed in duplicate and independently by two reviewers (M.K. and A.S.). Disagreements regarding the inclusion of studies for data extraction were resolved by the senior author (A.K.).

### 2.3 Data extraction

Duplicate work was removed after a final version of included literature was entered on excel sheets. We gathered available data on the origin of the reported case (country), date of publication, study type, relevant case within every included study, patient characteristics, age, sex, the status of Guillain‐Barré syndrome (present or absent), affected side of the face, the onset of facial palsy, the test used for detection of COVID‐19, features related to facial palsy, results of cerebrospinal fluid (CSF) analysis, COVID‐19 related symptoms, other signs/symptoms, imaging results, treatment regimen and outcome of treatment. Due to a lack of uniformity in the assessment of facial symptoms between included studies, the percentage‐wise prevalence of symptoms could not be calculated. Additionally, since follow‐up time varied between studies, treatment follow‐up results are not comparable. The terms “complete recovery,” “partial recovery,” “progressive improvement,” and “significant improvement” were regarded as positive treatment outcomes by the author of this review.

### 2.4 Quality assessment

The quality of included cases was assessed using Joanna Briggs institute's critical appraisal tools.^11^ Selected studies were examined for inclusion criteria, sample size, description of study participants, and setting. Two reviewers independently assessed the methodological quality of each paper. Quality assessments were done with different tools based on different study designs. Each tool was modified to provide a numeric score. Tools had 8 items for case reports and 10 for case series. Included case reports (*n* = 39) had a mean score of 6.385 ± 1.41 with scores ranging from 2 to 8^12^, ^13^, ^14^, ^15^, ^16^, ^17^, ^18^, ^19^, ^20^, ^21^, ^22^, ^23^, ^24^, ^25^, ^26^, ^27^, ^28^, ^29^, ^30^, ^31^, ^32^, ^33^, ^34^, ^35^, ^36^, ^37^, ^38^, ^39^, ^40^, ^41^, ^42^, ^43^, ^44^, ^45^, ^46^, ^47^, ^48^, ^49^, ^50^ 10 case series had a mean score of 5.60 ± 2.01, and scores ranged from 3 to 9.^51^, ^52^, ^53^, ^54^, ^55^, ^56^, ^57^, ^58^, ^59^ The detailed results of the quality assessment are provided in Supporting Information: File S1. The quality of our systematic review was assessed using AMSTAR 2 criteria.^60^ The level of compliance with AMSTAR 2 came out to be “low.” We could not conduct a meta‐analysis because only case reports and case series were included in the analyses without quantitative data.

### 2.5 Statistical analysis

This systematic review reported descriptive information using individual‐level data of 75 cases from a total of 49 studies reported on facial palsy as a manifestation of COVID‐19. The data focused on the date and country of publication, patient's characteristics, detailed symptoms of facial palsy and COVID‐19, the status of Guillain‐Barré syndrome (present or absent), results of imaging and Cerebrospinal Fluid (CSF) analysis, treatment plan and its outcome. In addition, the continuous variable's mean, median, and SD were calculated where possible.

## 3 RESULTS

**Figure 1:**
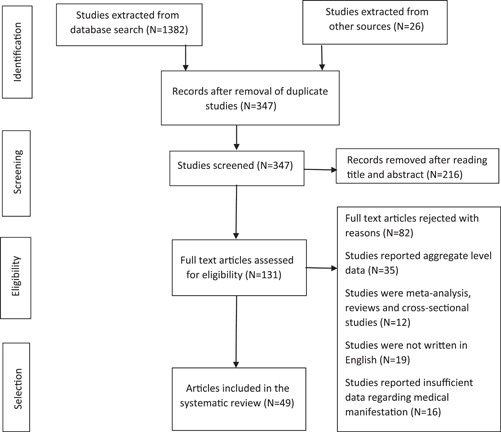
Flow chart of selected studies

Our initial search provided 1408 results. After removing duplicate studies (*N* = 1006), 347 studies were screened individually by the two reviewers (M.K. and A.S.). Two hundred and sixteen studies were rejected after going through their titles and abstracts, while full‐text versions of the remaining articles (*N* = 131) were opened to ascertain their relevance to the topic. Out of these, 82 were excluded for reporting aggregate‐level data (*N* = 35), not being of the desired study type (*N* = 12), not being in English (*N* = 19), or for reporting insufficient data on medical manifestation (*N* = 16). Finally, 49 studies met our inclusion criteria and were, thus, included for systematic analysis (Figure 1).

### 3.1 Patient characteristics

A total of 75 patients gathered from 49 studies who developed facial palsy due to COVID‐19 [Table 1]. The mean age of patients was 42.91 ± 19.59 years, ranging from 15 months to 88 years. In addition, 40 out of 75 patients were males. At the same time, 35 were females, giving a slightly higher ratio of male to female sufferers (8:7). Highest number of cases were reported from Brazil (*n* = 14), followed by the USA (*n* = 9), Turkey (*n* = 9), and Spain (*n* = 7). Iran and Italy reported six cases each, whereas Singapore, Morocco, and France published accounts of three people each suffering from the condition. India, Egypt, and Japan reported two cases each. At the same time, Canada, Nepal, UK, Belgium, Qatar, Germany, Sweden, Norway, and Portugal each had one published account of a patient complaining of facial palsy due to COVID‐19. It is noteworthy that 13 out of 62 patients had hypertension (20.97%), 9 had diabetes mellitus (14.52%), and 3 of the women were pregnant (4.84%). Twenty‐seven patients had no comorbid condition (43.55%), while no information was shared for 13 patients (17.33%).

**Table 1:** Patient characteristics

Author	Country	Study type	No of cases	Patient No.	Patient characteristics	Age/Sex	GB present	Affected side of face	Facial palsy as first sign or not	Features related to facial palsy	CSF results	COVID‐19 related symptoms	Other signs/symptoms	Imaging	Treatment	Treatment outcome	
Lima et al.	Brazil	Case series	8	1	None	43/F	No	Right	Yes	Moderate (HB Grade 3)	NS	Mild symptoms	Ipsilateral abducent nerve palsy	CT Scan normal	Oral corticosteroids	PR	
				2	None	25/F	No	Right	Yes	Mild (HB Grade 2)	NS	Mild	None	Brain MRI normal	Oral corticosteroids + acyclovir	CR.	
				3	None	33/F	No	Right	Yes	Moderate (HB Grade 3)	NS	Mild	None	NA	Oral corticosteroids + acyclovir	PR	
				4	None	26/F	No	Left	No (after 2–10 days for all Nos)	Mild (HB Grade 2)	NS	Mild	None	MRI: left CN7 enhancement	Oral corticosteroids	CR	
				5	None	50/F	No	Left	No	Moderate (HB Grade 3)	Protein: mildly elevated; WBC: normal; SARS‐COV: negative	Mild	None	CT scan: normal	Oral corticosteroids	PR	
				6	None	38/F	No	Left	No	Mild (HB Grade 2)	NS	Mild	None	Brain MRI: normal	Supportive (eye lubricant)	CR	
				7	None	39/F	No	Right	No	Mild (HB Grade 2)	NS	Mild	None	Brain MRI: normal	Oral corticosteroids	CR	
				8	None	34/M	No	Left	No	Mild (HB Grade 2)	NS	Mild	None	Brain MRI: normal	IV corticosteroids	CR	
Homma et al.	Japan	Case report	1	1	Smoker	35/F	No	Right	Yes	NA	NS	Cough, malaise, sore throat, nausea, fever, right‐sided aguesia of tongue and anosmia	None	CT scan: multiple bilateral ground‐glass opacities	Acetaminophen, Maoto, favipiravir, and inhaled Ciclesonide (corticosteroid)	CR	
Goh et al.	Singapore	Case report	1	1	NA	27/M	No	Left	No (after 6 days)	Left‐sided otalgia	NS	Myalgia, cough, fever, dysguesia, left‐sided throbbing headache, and conjunctival infection	None	Chest X‐ray: unremarkable; brain MRI; left CN7 enhancement	Oral corticosteroid, valacyclovir and Lopinavir/ritonavir	No significant improvement.	
Figueiredo et al.	Portugal	Case report	1	1	Pregnant	35/F	No	Left	Yes	Involuntary drooling, left‐side labial commissure deviation and ipsilateral lagophthalmos	NA	None	None	NA	Corticosteroid therapy and eye hydration therapy	No significant improvement.	
Caamaño et al.	Spain	Case report	1	1	None	61/M	Yes	Bilateral	No (after 10 days)	Involuntary drooling on his right facial commissure, unresponsive blink reflex on both eyes	Protein: mildly elevated; WBC: normal; SARS‐COV: negative	Fever, cough, and pneumonia	None	Brain MRI: unremarkable; Chest X‐ray: bilateral frosted glass pneumonia	Oral corticosteroid, antimalarial and lopinavir/Ritonavir	No significant improvement.	
Muras et al.	Spain	Case report	1	1	None	20/M	No	Bilateral	No (after a week)	NA	Protein: elevated; WBC: elevated; SARS‐COV: negative	Fever, significant asthenia, headache, myalgia, nausea, headache, odynophagia and vomiting	EBV coinfection	Brain MRI: confirmed bilateral facial neuritis	Levofloxacin and oral corticosteroid	CR	
Manganotti et al.	Italy	Case series	3	1	NA	72/M	Yes	Right	No (after 18 days)	Mild right sided lower face weakness	Protein: elevated; WBC: normal; SARS‐COV: negative	Fever, dyspnea, hyposmia, ageusia	Flaccid tetraparesis, hypesthesia of extremities, dysuria, dysphasia, sinus arrythmia	NA	IVIG cycle, antimalarial, oseltamivir, darunavir, IV corticosteroid, and tocilizumab	NA	
				2	NA	49/F	Yes	Right	No (after 14 days)	Right‐sided hypoesthesia of the face	Protein: elevated; WBC: normal; SARS‐COV: negative	Fever, cough, dyspnea, hyposmia, and ageusia	Ophthalmoplegia with diplopia in the vertical and lateral gaze, and limb ataxia	Brain MRI: normal	IVIG cycle, antimalarial, lopinavir–ritonavir, IV corticosteroid	Progressive improvement	
				3	NA	76/M	Yes	Left	No (after 22 days)	Mild left‐sided lower facial deficit	Protein: elevated; WBC: normal; SARS‐COV: negative	Fever, cough, hyposmia, ageusia	Mild transient diplopia, tetraparesis and dysuria	NA	IVIG cycle, oseltamivir, darunavir, IV corticosteroid,	Progressive improvement	
															Tocilizumab,		
															meropenem, linezolid		
															clarithromycin,		
															doxycycline and fluconazole		
Khaja et al.	USA	Case report	1	1	HTN and asthma	44/M	Yes	Bilateral	No (after 3 days)	Severe (HB Grade 5)	Protein: elevated; WBC: normal; glucose: normal	Ageusia	None	Chest X‐ray: clear; MRI brain: unremarkable	IVIG	CR	
Sancho‐Saldaña et al.	Spain	Case report	1	1	None	56/F	Yes	Bilateral	No (after 20 days)	NA	Protein: elevated; WBC: normal; SARS‐COV: negative	Fever, dry cough, and dyspnea	Tetraparesis, lumbar pain, pararesthesia in both hands and oropharyngeal weakness	Chest X‐ray: lobar consolidation	Antimalarial, azithromycin and IVIG	PR	
Theophanous et al.	USA	Case report	1	1	Prematurely born, multiple congenital abnormalities, asthma, and gastrostomy tube feeding	6/M	No	Right	Yes	Moderate severe (HB Grade 4)	NA	None	Tachycardiac	NA	IV acyclovir, IVIG infusion, lubricating eye drops and IV corticosteroids	Significant improvement	
Dahl et al.	Norway	Case report	1	1	Acute MI	37/M	No	Right	No (after 18 days)	NA	Protein: elevated; IgG: normal; WBC: elevated	Fever, headache, dyspnea	Oliguria, hypotension, tachycardiac, tachypneic and unilateral painful neck swelling	X‐ray thorax: bibasal consolidations	IV furosemide and intermittently required low‐dose norepinephrine	CR	
Egilmez et al.	Turkey	Retrospective cohort	8	1	HTN, CHF	90/M	No	Left	Yes	Moderate severe (HB Grade 4)	NA	Pneumonia	None	Thorax CT: Intense pneumonia with ground glass opacities	IV moxifloxacin and corticosteroids (dexamethasone and prednisolone)	PR	
				2	None	4/F	No	Left	No (after 7 days)	Moderate severe (HB Grade 4)	NA	Cough and fever	None	Thorax CT: normal	Oral corticosteroid	CR	
				3	None	17/F	No	Right	Yes	Moderate (HB Grade 3)	NA	Cough, ageusia and anosmia	None	Thorax CT: normal	Favipravir and oral corticosteroid	CR	
				4	HTN, DM	71/F	No	Right	Yes	Moderate severe (HB Grade 4)	NA	Fever, ageusia and anosmia	None	Thorax CT: normal	Favipravir and IV corticosteroid	CR	
				5	None	63/F	No	Left	Yes	Moderate severe (HB Grade 4)	NA	Fever, myalgia, ageusia and anosmia	None	Thorax CT: Mild pneumonia with ground glass appearance	Favipravir and oral corticosteroid	PR	
				6	None	60/F	No	Left	No (after 12 days)	Moderate severe (HB Grade 4)	NA	Fever, ageusia and anosmia	None	Thorax CT: normal	Favipravir and oral corticosteroid	PR	
				7	HTN	65/F	No	Left	Yes	Moderate (HB Grade 3)	NA	Ageusia and anosmia	None	Thorax CT: Mild pneumonia with ground glass opacities	IV corticosteroids	PR	
				8	HTN, OSA	30/M	No	Left	No (after 9 days)	Moderate (HB Grade 3)	NA	ageusia and anosmia	None	Thorax CT: Mild pneumonia with ground glass appearance; brain MRI: normal	Favipravir and oral corticosteroid (methylprednisolone and dexamethasone)	No improvement	
Engström et al.	Sweden	Case report	1	1	None	46/F	No	Left	No (after 26 days)	Tongue deviation to left, inability to wrinkle forehead and left lagophthalmos, drooping left corner of mouth, vocal cord paresis, left‐sided paresis	NA	High fever, cough, dyspnea, dysphagia, and severe headaches	None	CT thorax: bilateral ground glass appearance. MRI brain: some edema in the parotid gland	High‐flow oxygen therapy, dalteparin, IV cefotaxime, oral and IV corticosteroids, and tear substitutes with watch bandages	Significant improvement	
Corrêa et al.	Brazil	Case series	4	1	None	25/F	No	Right	No (after 2 weeks)	Right‐sided facial muscle weakness and right lagophthalmos	NA	Vertigo, mild dyspnea, and fever	Strabismus in the right eye after right CN6 palsy	Brain MRI: restricted diffusion (right CN6 nucleus) and an asymmetrical enhancement (right CN7)	Oral corticosteroids	CR	
				2	None	30/F	No	Right	No (after 10 days)	NA	NA	Mild fever and sore throat	None	Brain MRI: enhancement in right CN7	Oral corticosteroids	CR	
				3	OA, AF	65/M	Yes	Bilateral	No (after 2 weeks)	NA	Protein: elevated; WBC: normal; SARS‐COV: negative	Headache, fever, and generalized myalgia	Lower limbs weakness	Brain MRI: bilateral enhancement in CN7	IVIg	PR	
				4	None	33/M	No	Bilateral	No (after 2 weeks)	NA	NA	Fever	NA	Brain MRI: enhancement in CN7	Oral corticosteroids	CR	
Chan et al.	Canada	Case report	1	1	None	58/M	Yes	Bilateral	Yes	Dysarthria, bilateral lagophthalmos, inability to raise eyebrow, wrinkle forehead, smile, and close lips	Protein: elevated; WBC: normal; SARS‐COV: negative	None	Hypertension, tachypnea, paresthesia in his feet, and tachycardia	Chest X‐ray: bilateral infiltrates; CT: bilateral ground‐glass opacities in lung apices; Brain MRI: Bilateral CN7 enhancement	Empiric ceftriaxone, azithromycin and IVIG	PR	
Decio et al.	Italy	Correspondence	1	1	NA	1.25/F	No	Right	NA	NA	NA	Mild respiratory symptoms, fever, anosmia, and ageusia	None	Brain MRI: right CN7 enhancement	Oral corticosteroids	CR	
Ozer et al.	Turkey	Case report	1	1	NA	62/F	No	Left	No (after 2 days)	Total paralysis (HB Grade 6)	NA	Fatigue, chills, and myalgia	Sensorineural hearing loss	Brain MRI: CN7 and CN 8 enhancement	Oral corticosteroids, famotidine, oral favipiravir and SQ enoxaparin sodium	PR	
Neo et al.	Singapore	Case series	2	1	NA	25/M	No	Left	Yes	Severe (HB Grade 5)	NA	None	None	All imaging were unremarkable	Oral corticocorticosteroids, valaciclovir and given eye care advice	CR	
				2	NA	34/M	No	Right	Yes	Moderate severe (HB Grade 4)	NA	None	None	All imaging were unremarkable	Oral corticocorticosteroids, valaciclovir and given eye care advice	PR	
Mackenzie et al.	USA	Case report	1	1	HTN, T2DM	39/F	Yes	Bilateral	No (after 20 days)	NA	NA	Ageusia, anosmia, headache, myalgias, malaise, and cough	Left arm paresthesia, generalized flaccid areflexia, and inability to walk	NA	Enoxaparin SC, losartan, meperidine IV, antimalarial drug, oral corticosteroids and plasmapheresis	PR	
Bastola et al.	Nepal	Case report	1	1	DM	48/M	No	Left	No (after 4 days)	Left‐sided facial droop with inability to wrinkle left forehead, raise left eyebrow and left laogphthalmus	NA	Mild dry cough and hyposmia	None	HRCT chest: ground‐glass opacity in the right lower lobe	Regular insulin and other antidiabetic medications, tear plus drops for dry eyes, and IV corticosteroid	Significant improvement with some residual weakness	
Hookham et al.	UK	Case report	1	1	Childhood asthma and HTN	17/M	No	Right	No (after 1.5 months)	Right‐sided facial droop with right‐sided facial hypoesthesia	NA	Fever, diarrhea, vomiting, mild headache, intermittent right‐sided chest pain, myalgia and lethargy, diaphoretic and conjunctival injection (anterior uveitis)	Pediatric inflammatory multisystem syndrome, tachycardiac, tachypnea, raised blood pressure, palpitations	Brain MRI: minimal increased enhancement of a segment of right CN 7	IV fluids, broad spectrum antibiotics, oral corticosteroids, tocilizumab, amlodipine (for HTN), aspirin and eye drops	NA	
Khedr et al.	Egypt	Case report	2	1	None	49/F	Yes	Left	No	Right‐sided deviation of mouth and left lagophthalmos	NA	Fever, dysphagia, and vomiting	Flaccid areflexic quadriplegia, hoarseness of voice, and an impaired cough reflex and stock and glove hypoesthesia	CT chest: bilateral ground‐glass opacities	Plasmapheresis and IVIg	Progressive improvement	
				2	None	55/F	Yes	Bilateral	No	Bilateral inability to close eyes, with reduced blinking, inability to whistle, protrude the lips or expose the teeth.	NA	Fever, cough, and expectoration	flaccid areflexic quadriplegia, stock and glove hypoesthesia	CT chest: bilateral ground‐glass opacities	IVIg	CR	
Kumar et al.	India	Case report	1	1	pregnant and PCOS	28/F	No	Right	No	Inability to wrinkle right forehead and close right eye, left‐sided deviation of mouth, numbness of the right side of the face and right‐sided drooling	NA	Fever, dysgeusia, and anosmia	Persistently high blood pressure (160/110), generalized weakness	NA	Oral valacyclovir and oral corticosteroid, insulin (for steroid‐induced DM) with physiotherapy and eye protective measures	CR	
Aasfara et al.	Morocco	Case report	1	1	Pregnant	36/F	Yes	Bilateral	Yes	Moderate severe (HB Grade 4)	protein: elevated; WBC: normal cell count; glucose: normal	Vertigo, nausea, and vomiting	asymmetric numbness in the lower limbs and left fingers, right sensorineural hearing loss, right vestibular areflexia and nystagmus	NA	IVIg and IV corticosteroids	CR of facial palsy.	
Paybast et al.	Iran	Case report	1	1	HTN	38/M	Yes	Bilateral	Yes	Bilateral facial droop, drooling, and slurred speech	Glucose: normal; WBC: normal; protein: elevated	Band‐like headache, dysphagia, and mild dizziness	quadriparesthesia, decrease in all sensation modalities in four limbs affecting the distal parts up to ankle and elbow joints, tachycardia, blood pressure instability	NA	Plasmapharesis and labetalol (for HTN)	No significant improvement	
Bigaut et al.	France	Case report	2	1	None	43/M	Yes	Right	No	NA	WBC: normal; protein: elevated	Cough, anosmia, ageusia, and diarrhea	Flaccid paraparesis, generalized areflexia, hypoesthesia, fore limb paresthesia, ataxia, myalgias in legs	Chest CT: bilateral ground‐glass opacities; MRI: CN 3, 5, 6, 7, and 8 neuritis	IVIg	Progressive improvement	
				2	Obesity	70/M	Yes	Left	No	NA	WBC: normal; protein: elevated	anosmia, ageusia, diarrhea, dyspnea	Flaccid tetraparesis, generalized areflexia, forelimb paresthesia	Chest CT: bilateral moderate ground‐glass opacities	IVIg and physiotherapy	No significant improvement.	
Ottaviani et al.	Italy	Case report	1	1	Mild HTN	66/F	NA	left	Yes	NA	Protein: elevated; rest; normal	Acute fatigue, mild fever, and cough	Paraplegia, transient pruriginous dorsal rash, initial distal weakness in the upper limbs and diffuse areflexia	Lung CT: bilateral ground‐glass opacities	IVIg, lopinavir/ritonavir and antimalarial	NA	
Casas et al.	Spain	Case report	1	1	vWB	32/M	No	Left	No	Moderate severe (HB Grade 4)	NA	Malaise, fever, dry cough, and headache	None	Brain MRI: asymmetric contrast uptake in a segment of Left CN7	acetaminophen, metamizole, physiotherapy and ocular hydration	CR.	
Hutchins et al.	USA	Case report	1	1	HTN, prediabetes, Class I obesity	21/M	Yes	Bilateral	No	Dysarthria, hypogeusia, and facial numbness	Protein: mildly elevated; WBC: normal; SARS‐COV: negative	Fever, cough, dyspnea, diarrhea, nausea, headache, and sinonasal congestion, dizziness, hypogeusia	Tachycardic, bilateral lower extremity weakness, bilateral upper extremity paranesthesia, Grade 4/5 weakness in the deltoids and hip flexors bilaterally, diffuse areflexia	Chest X‐ray: increased bilateral air space opacities; brain MRI: abnormal bilateral enhancement of CN 6 and 7, alongside right CN 3	Plasmapheresis	Nonsignificant improvement	
Abolmaali et al.	Iran	Case series	3	1	HTN	88/F	Yes	Left	Yes	Left lagophthalmos and neck flexion weakness	Protein: elevated; rest: normal	Fatigue	Quadriparesis, low back and thigh pain, impaired proprioception	CT: pneumonia with a ground‐glass pattern	Plasmapharesis, intubation, corticosteroids, antimalarial and lopinavir/ritonavir	No significant improvement.	
				2	NA	47/M	Yes	Bilateral	No	Weakness of neck flexors and dysarthria	Protein: elevated; rest: normal	Fatigue, dyspnea, and cough	Generalized hyporeflexia, urinary retention, quadriparesis, low back pain	CT: ground‐glass opacities	Plasmapharesis, intubation, corticosteroids, antimalarial and lopinavir/ritonavir	Death	
				3	NA	58/M	Yes	NA	No	NA	Protein: elevated; rest: normal	Progressive dyspnea, dry cough, and dizziness	Muscle weakness, gait disturbance and areflexia.	CT: ground‐glass opacities	Plasmapharesis, IVIg, remdesivir, antimalarial, favipiravir and lopinavir/ritonavir	Death	
Oke et al.	USA	Case report	1	1	history of nephrolithiasis	36/M	No	Right	No	Moderate severe (HB Grade 4)	NA	Fever and body aches	NA	Brain MRI: asymmetric enhancement of the right CN7	Oral valacyclovir, corticosteroid, eye patch and artificial tears	Significant improvement	
Derollez et al.	France	Case report	1	1	Overweight	57/F	NA	Left	No	NA	NA	Fatigue, myalgia, chills, and moderate cough	NA	Chest X‐ray: infiltrates	Ocular protection	CR	

Hasibi et al.	Iran	Case report	1	1	Class 1 obesity	52/M	No	Right	No	Severe (HB Grade 5)	NA	Fever, malaise, dry cough, and anorexia	NA	CT: multiple bilateral peripheral ground glass opacities	Oral and corticosteroid, favipiravir, remdesivir, arbidol and NSAID	CR	
Taouihar et al	Morocco	Case report	2	1	DM, CML	39/M	No	Right	Yes	Facial asymmetry, dysarthria, and difficulty chewing	NA	Dyspnea	NA	NA	Azithromycin, zinc, vitamin C, oral corticosteroid, preventive anticoagulation	CR of facial palsy	
				2	DM, HTN	57/M	No	Right	Yes	Dysarthria, facial asymmetry, swallowing disorder, and left‐sided deviation of mouth	NA	Dyspnea	NA	NA	Azithromycin, zinc, vitamin C, oral corticosteroid, and preventive anticoagulation	Significant improvement	
Kaplan et al.	USA	Case report	1	1	DM	48/F	No	Left	No	Asymmetric forehead folds, dry eye, inability to raise the left eyebrow and left facial droop	NA	Fever, chills, headaches, fatigue, myalgia, and weakness	NA	CT: bilateral ground‐glass opacities	Oral corticosteroids, valacyclovir, and doxycycline	Significant improvement	
Kerstens et al.	Belgium	Case report	1	1	NA	27/M	No	Bilateral	Yes	Severe (HB Grade 5)	IgG: elevated; rest: normal	Ageusia	None	MRI: bilateral CN7 contrast enhancement	Valaciclovir, artificial tears and oral corticosteroids	CR	
Kakumoto et al.	Japan	Case report	1	1	NA	22/M	Yes	Bilateral	No	Dysarthria	Protein: elevated; rest: normal	Fever and dysphagia	Tetraparesis, hypesthesia of extremities, dysuria, inability to defecate, dyschezia, sinus arrythmia.	Head MRI: bilateral CN7 contrast enhancement	IVIG, intubated and managed on a ventilator	CR	
Al‐Mashdali et al.	Qatar	Case report	1	1	Atrial septal defect	21/M	No	Right	No	NA	NA	Fever, cough, watery diarrhea, vomiting, conjunctivitis, and abdominal pain	Acute myocarditis	CT: Bilateral ground‐glass opacities and pleural effusion	IV corticosteroids and ocular lubricant	Significant improvement	
Judge et al.	USA	Case report	1	1	NA	64/M	No	Bilateral	No	Dysarthria and subjective facial paresthesia	WBC: elevated; protein: elevated; Glucose: normal	Cough, fever, and chills	None	NA	NA	Progressive improvement	
Tran et al.	USA	Case report	1	1	DM	42/M	Yes	Right	Yes	Right‐sided hypesthesia, dysarthria, diplopia, ptosis, and inability to raise eyebrows or smile	protein: elevated; WBC: normal; glucose: elevated	None	Right lower extremity weakness	Chest X‐ray: bibasilar infiltrates; CT: ground‐glass opacities	IV corticosteroids, electrolyte replacement for hypokalemia, IVIg, physical, occupational, and speech therapy	CR	
Silveira et al.	Brazil	Case report	1	1	DM, HTN	65/M	No	Left	Yes	Facial asymmetry, otalgia, and ophthalmoplegia	NA	Fever, dry cough, and dyspnea	Clear left eye proptosis and blindness, otorrhea and complete hearing loss on the left and partial hearing on the right	Initial CT: erosion of the anterior wall of the left external ear conduct and left mandible condyle; brain MRI: compression of left CN 2, 3, 4 and 6	IV Meropenem, IV vancomycin, IV Ciprofloxacin, and mastoidectomy	Death	
Liberatore et al.	Italy	Case report	1	1	HTN and history of testicular seminoma	49/M	Yes	Left	No	NA	Glucose: normal; protein: slight elevation; WBC: normal	Fever, cough	Symmetric weakness in the upper limbs with flaccid tone, reduced tendon reflexes, and respiratory insufficiency; gastroparesis, alternating episodes of tachy‐/bradyarrhythmia, and frequent hypertensive crises)	Chest CT: multifocal ground‐glass opacities; Brain MRI: normal	Antimalarial, lopinavir/ritonavir, and ceftriaxone	NA	


Shinde et al.	India	Case report	1	1	HTN	64/M	No	Right	Yes	Severe (HB Grade 5)	NA	None	Macular erythematous rash along zygomatic arch, maxillary and mandibular division of trigeminal nerve	Chest X‐ray: normal	Eye care, acyclovir, corticosteroid, and methyl cobalamin	PR	
Ochoa‐Fernández et al.	Spain	Case report	1	1	None	6/F	No	Left	Yes	Moderate (HB Grade 3)	NA	None	None	NA	Eye protection and oral corticosteroids	CR	
Zain et al.	USA	Case report	1	1	None	2/F	No	Right	Yes	Right lagophthalmos, ptosis, and drooping of corner of mouth, flattening of the nasiolabial fold, dryness of the eye and tearing	Glucose: normal; protein: normal; WBC: normal	None	EBV coinfection and contact dermatitis	Brain MRI: abnormal enhancement of the canalicular segment of right CN7	IV corticosteroids	CR	
Ribeiro et al.	Brazil	Case report	1	1	None	26/M	No	Right	No (on 8th day from first onset of symptoms)	Right facial weakness	NA	Cough and fever	None	Chest CT: multiple bilateral ground‐glass opacities and some superimposed intralobular septal thickening; Brain MRI: enhancement of the right CN7	NA	NA	
González‐Castro et al.	Spain	Case series	2	1	Obesity	40/F	No	Left	No (after 2nd day of ward admission)	Moderate (HB Grade 3)	NA	NA	None	MRI: poorly defined contrast uptake in the left hemifacial/malar subcutaneous region	High‐flow oxygen therapy	NA	
				2	DM, smoker and Parkinson's disease patient	65/M	No	Left	No	Moderate (HB Grade 3)	NA	NA	None	NA	High‐flow oxygen therapy	NA	
Pelea et al.	Germany	Case report	1	1	HTN, hypothereosis	56/F	yes	Bilateral	No	Severe (HB Grade 5)	Protein: elevated; glucose: normal; WBC: elevated; SARS‐COV: negative	Dry cough, mild fever, and a general weakness	Tingling sensation in all fingertips and toes, flaccid tetraparesis, arreflexia and tachycardia	Chest CT: leaky infiltrates in the right lower lobe	IVIG and Plasmapharesis	PR	
Karimi‐Galougahi	Iran	Letter to the editor	1	1	None	60/M	No	Right	No	Right‐sided facial nerve palsy, involving mouth, eye, and forehead	NA	Fever, cough, and dyspnea	None	Chest CT: ground‐glass opacitiesAbbr	Remdesivir, corticosteroid, and oxygen therapy	NA	

Abbreviations: CR, complete recovery; CT, computed tomography; IVIg, intravenous immunoglobulin; MRI, magnetic resonance imaging; NA, not applicable; PR, partial recovery.

### 3.2 Symptom presentation

Noticeably, 30.14% of the patients were diagnosed with Guillain‐Barré Syndrome associated with COVID‐19. Facial palsy was observed as an initial symptom in 26/74 (35.14%) patients. In all, 22.97% of patients complained of bilateral facial paralysis, whereas ipsilateral paralysis was observed in 77.03%. Out of this 77.03% of patients, left (*n* = 29) and right (*n* = 28) sided involvement was observed in an almost equal number of patients. In all, 76.47% of bilateral facial paralysis patients also had GBS. Varying intensity of facial paralysis was seen among the 75 COVID‐19‐inflicted patients. While some experienced only mild facial deficit, weakness, or hypesthesia of the face, others complained of complete facial paralysis (*n* = 1). Most people complained of moderate‐severe facial dysfunction (*n* = 10) based on the House Brackmann scale. Among sufferers, Lagophthalmos, otalgia, facial drooping, dysarthria, and compromised forehead wrinkling were common complaints. Most patients witnessed mild to moderate COVID‐19‐specific symptoms. These included complaints of fever, fatigue, cough, ageusia, and headache.

### 3.3 Diagnostic results

Polymerase chain reaction (PCR), reverse transcriptase (RT)‐PCR, and serology were the most used tests to confirm COVID‐19 infection among 75 individuals. CSF analysis was performed in only half (48%) of the patients, 55.56% of whom had elevated CSF protein levels. CSF SARS‐CoV‐2 result was negative in all (100%), while approximately 1 in every 7 patients (13.5%) had elevated WBC levels on CSF report. Moreover, some patients underwent radiologic imaging to reach a diagnosis. Common findings on chest X‐ray, computer tomography (CT) thorax, and magnetic resonance imaging (MRI) brain included the presence of infiltrates/consolidations (50%), ground glass opacities (71.88%), and enhancement of CN 7 of the affected side (51.52%), respectively.

### 3.4 Treatment regimen and disease prognosis

Data on treatment plans were shared for 73 (97.33%) out of 75 patients. Although every individual had a treatment plan tailored according to his age, comorbidities, severity of the condition, availability of resources, and so forth, a handful of overlapping medications were prescribed too. Treatment regimen mainly included the use of corticosteroids (69.86%), antivirals (31.51%), IVIG (24.66%), antibiotics (17.81%), antiretroviral (12.33%), and antimalarial (10.96%) medications. Lopinavir/Ritonavir was the most readily prescribed antiretrovirals, whereas hydrochloroquine (100%) was the only antimalarial advised to patients. Three antivirals, namely, acyclovir, valacyclovir, and favipiravir, were predominantly administered to these patients. In total, 35.62% of patients (*n* = 26) adhered to a combination of antiviral‐corticosteroid‐based therapy. Furthermore, in some cases, eye care (19.18%) medications, for example, lubricants, artificial tears and watch bandages, and so forth, were also encouraged eye care. Physiotherapy (5.48%) and plasmapheresis (10.96%), though less common, were also a part of the treatment plan of some patients. The outcome of treatment was provided for 67 (89.33%) patients. Positive treatment outcomes were observed in 83.58% of cases. In contrast, 10 patients (14.93%) showed nonsignificant recovery, out of which 3 (4.48%) died from the disease.

## 4 DISCUSSION

Systematic reviews have been conducted in the past that discussed the association of facial palsy with COVID‐19. The reviews indirectly discussed facial palsy concerning COVID‐19 by establishing the correlation of COVID‐19 with GBS, while some only explored cases in which facial palsy was an isolated neurological finding.^8^, ^61^, ^62^ However, none of them collectively assessed all the cases of facial palsy seconCarrillodary to COVID‐19. In our study, we could summate the vast amount of literature available by including all the cases of peripheral facial palsy secondary to COVID‐19 regardless of associated conditions. Thus, we were able to collate a stronger evidence base to support our findings concerning facial palsy and COVID‐19.

Cranial nerve involvement in GBS most commonly results in bilateral facial palsy, rarely unilateral involvement, and facial palsy mostly occurs in the early stage of the disease.^63^, ^64^ The findings of our study corroborate this observation. Of the 17 patients who demonstrated bilateral facial nerve palsy, 13 (76.5%) had accompanying GBS. Thus, cases of bilateral facial palsy are mostly attributed to GBS.

### 4.1 Pathophysiology

**Figure 2:**
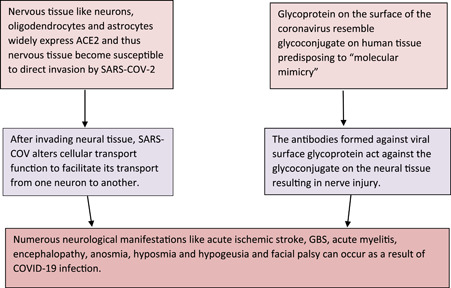
Pathophysiology behind the onset of facial palsy in COVID‐19 patients

Pathomechanisms of nervous tissue involvement have been discussed in great detail in the literature.^65^, ^66^, ^67^, ^68^, ^69^, ^70^, ^71^ Some of these mechanisms have been highlighted in Figure 2. In our study, unilateral facial palsy patients demonstrated right and left side involvement in almost equal proportions. This shows that SARS‐CoV‐2 has an equivalent predilection for right and left facial nerves.

In clinical practice, various other viruses have been observed to be associated with facial palsy as well, which include echovirus, enterovirus, herpes simplex virus, Epstein‐Barr virus, cytomegalovirus, human herpesvirus 6, human immunodeficiency virus, mumps, rubella, poliomyelitis, and varicella zoster virus.^72^, ^73^, ^74^ Thus, this further corroborates that a virus like SARS‐CoV‐2 could be behind the etiopathogenesis of facial palsy.

Classically, facial palsy is known to show a predominance in females, which is evident in the prevalence studies conducted.^66^, ^75^ Moreover, a systematic review involving studies in which Bell's palsy was the only major neurological manifestation in COVID‐19 patients also showed a female preponderance.^8^ However, our study demonstrated a slightly high male preponderance. This is because our study included a significantly high number (30.14%) of patients with accompanying GBS, and previous reviews evaluating the relationship between COVID‐19 and GBS have demonstrated a high male preponderance.^61^, ^62^, ^76^ In addition, approximately 21% of the patients had hypertension, and 14.5% demonstrated diabetes mellitus. This finding is corroborated by a study conducted in Korea which demonstrated that facial palsy was associated with age, gender, smoking status, alcohol drinking, history of hypertension, stroke, CVD, diabetes mellitus, total cholesterol level in the blood, and hearing loss through a univariable analysis.^75^

Furthermore, Paolino and colleagues^77^,^78^ reported a greater frequency of arterial hypertension and lipid disorders in patients with Bell's palsy than in controls. Moreover, a study showed that the risk of Bell's palsy was increased in diabetes.^79^ Also, patients with underlying comorbidities such as DM, obesity, hypertension, respiratory distress, or advanced age are at higher risk of developing COVID‐19.^80^ Thus, all these factors contribute to the findings of our study.

The treatment regimen mainly involved corticosteroids (69.86%), and 35.62% of patients adhered to a combination of antiviral‐corticosteroid‐based therapy. This finding corroborates a meta‐analysis demonstrating significant benefits of treating Bell's palsy with corticosteroids.^81^ Moreover, a network meta‐analysis showed that combined therapy remains the best regimen for a good recovery outcome, supporting its use by a significant 35.62% of patients.^82^ However, only two patients used antivirals without corticosteroids to treat facial palsy. A systematic review supports this finding by demonstrating that corticosteroids alone were superior to antivirals alone in treating facial palsy. There was no clear benefit from antivirals alone over placebo.^83^ Our findings show that the successful regimens in treating facial palsy due to other etiologies are also effective in treating facial palsy secondary to COVID‐19.

Positive treatment outcomes were observed in 83.58% of patients. This corroborates the effectiveness of the treatment regimens used in the case reports to treat facial palsy secondary to COVID‐19. A favorable response to treatment has also been shown in other complications that arise secondary to COVID‐19, such as central retinal vein occlusion.^7^ However, some complications, such as hypoxic encephalopathy, have also shown a poor prognosis.^84^ Thus, this highlights that many distinct complications can arise due to COVID‐19 with differing pathogenesis and severity.

There were some limitations in our study. Due to lack of provision of pertinent analytical data, no meta‐analysis could be conducted on the topic to confirm the relationship between COVID‐19 and facial palsy. Our review only comprised of case reports/series in which a limited number of patients were assessed. Therefore, large‐scale studies with more patients and longer follow‐ups are warranted to reliably draw the correlation between COVID‐19 and facial palsy. Moreover, studies in languages other than English were excluded from the analyses. Lastly, adequate representation of most countries was not seen in our review, which implies that many cases went unreported there, so they could not be included in our analyses. Despite the limitations, we tried to include all relevant cases to date and demonstrated an in‐depth comparison of clinical, radiological, and diagnostic features of COVID‐19 and concomitant facial palsy in our patient‐level analyses.

## 5 CONCLUSION

To the best of our knowledge, this is the most updated review of facial palsy cases following COVID‐19 infection. Although our patient‐level systematic review successfully collated published accounts of facial palsy cases post COVID‐19 infection while theorizing the pathophysiology behind COVID‐19 and subsequent onset of facial palsy, the likelihood of the association being purely coincidental cannot be overlooked. Therefore, large‐scale studies are still warranted to thoroughly understand the association between COVID‐19 and concomitant facial palsy. Systematic reviews involving studies with large sample sizes, such as retrospective cohorts, should be conducted, generating a large patient pool for analyses. This would allow us to develop a clearer understanding of patient characteristics and devise more effective treatment regimens that cater to the needs of individual patients.

## AUTHOR CONTRIBUTIONS

**Aiman Khurshid**: Conceptualization, methodology, writing – original draft preparation, writing – review & editing. **Maman Khurshid, Aruba Sohail, Imran Mansoor Raza, Muhammad Khubab Ahsan, Mir Umer Farooq Alam Shah, Anab Rehan Taseer, Abdulqadir J. Nashwan, Irfan Ullah**: Data curation, writing – review & editing. All authors read and approved the final manuscript.

## CONFLICT OF INTEREST

Abdulqadir J. Nashwan is an Editorial Board member of Health Science Reports and co‐author of this article. He is excluded from editorial decision‐making related to the acceptance of this article for publication in the journal. The remaining authors declare no conflict of interest.

## TRANSPARENCY STATEMENT

The lead author Abdulqadir J. Nashwan affirms that this manuscript is an honest, accurate, and transparent account of the study being reported; that no important aspects of the study have been omitted; and that any discrepancies from the study as planned (and, if relevant, registered) have been explained.

## Supporting information

Supporting information.

## Data Availability

The authors confirm that the data supporting the findings of this study are available within the article and its Supporting Information.
