# Supplementary material for: Facial palsy as a manifestation of COVID‐19: A systematic review of cases
Source: Health Sci Rep. 2022 Oct 28;5(6):e887. doi: 10.1002/hsr2.887 (PMC9616168; doi:10.1002/hsr2.887)
Supplement: Supplementary file 1 — Supporting information. [file HSR2-5-e887-s001.docx]

Supplementary file

Search strategy for PubMed

1)COVID-19

"sars cov 2"[MeSH Terms] OR "sars cov 2"[All Fields] OR "sars cov 2"[All Fields] OR "covid 19"[MeSH Terms] OR "covid 19"[All Fields] OR "coronavirus disease 2019"[All Fields] OR "covid 19"[All Fields] OR "covid 19"[MeSH Terms] OR “covid 19 serotherapy"[All Fields] OR "covid 19 nucleic acid testing"[All Fields] OR "covid 19 nucleic acid testing"[MeSH Terms] OR "covid 19 serological testing"[All Fields] OR "covid 19 serological testing"[MeSH Terms] OR "covid 19 testing"[All Fields] OR "covid 19 testing"[MeSH Terms] OR "sars cov 2"[All Fields] OR "sars cov 2"[MeSH Terms] OR "severe acute respiratory syndrome coronavirus 2"[All Fields] OR "ncov"[All Fields] OR "2019 ncov"[All Fields] OR "coronavirus"[MeSH Terms] OR "coronavirus"[All Fields] OR "cov"[All Fields] OR "sars cov 2"[MeSH Terms] OR "sars cov 2"[All Fields] OR "severe acute respiratory syndrome coronavirus 2"[All Fields] OR "coronavirus infections"[MeSH Terms] OR ("coronavirus"[All Fields] AND "infections"[All Fields]) OR "coronavirus infections"[All Fields] OR ("coronavirus"[All Fields] AND "infection"[All Fields]) OR "coronavirus infection"[All Fields]

AND

2) facial palsy OR facial paresis OR facial deficits

"facial paralysis"[MeSH Terms] OR ("facial"[All Fields] AND "paralysis"[All Fields]) OR "facial paralysis"[All Fields] OR ("facial"[All Fields] AND "palsy"[All Fields]) OR "facial palsy"[All Fields] OR ("facial paralysis"[MeSH Terms] OR ("facial"[All Fields] AND "paralysis"[All Fields]) OR "facial paralysis"[All Fields] OR ("facial"[All Fields] AND "paresis"[All Fields]) OR "facial paresis"[All Fields]) OR (("face"[MeSH Terms] OR "face"[All Fields] OR "facial"[All Fields] OR "facials"[All Fields]) AND ("deficit"[All Fields] OR "deficits"[All Fields]))

3) Human

Filter

From 1^st^ December, 2019 to September 21, 2021.

Search results:

699

Joanna Brigg’s Institute Critical Appraisal Checklist for Case Reports.

| Author’s name | Q1 | Q2 | Q3 | Q4 | Q5 | Q6 | Q7 | Q8 | Total |
| --- | --- | --- | --- | --- | --- | --- | --- | --- | --- |
| Homma et al. [12] | Yes | No | Yes | Yes | Yes | Yes | No | Yes | 6/8 |
| Goh et al. [13] | No | Yes | Yes | Yes | No | Yes | Yes | Yes | 6/8 |
| Figueiredo et al. [14] | No | Yes | Yes | Yes | Yes | Yes | Yes | Yes | 7/8 |
| Caamaño et al. [15] | No | Yes | Yes | Yes | No | Yes | No | Yes | 5/8 |
| Muras et al. [16] | No | Yes | Yes | Yes | No | Yes | No | Yes | 5/8 |
| Khaja et al. [17] | No | Yes | Yes | Yes | Yes | No | No | Yes | 5/8 |
| Sancho-Saldaña et al. [18] | No | Yes | Yes | Yes | Yes | Yes | Yes | Yes | 7/8 |
| Theophanous et al. [19] | Yes | No | Yes | Yes | Yes | Yes | Yes | Yes | 7/8 |
| Dahl et al. [20] | No | No | Yes | Yes | Yes | Yes | Yes | Yes | 6/8 |
| Engström et al. [21] | No | Yes | Yes | Yes | Yes | Yes | Yes | Yes | 7/8 |
| Chan et al. [22] | No | Yes | Yes | Yes | Yes | Yes | Yes | Yes | 7/8 |
| Decio et al. [23] | No | No | No | Yes | Yes | No | No | No | 2/8 |
| Ozer et al. [24] | No | Yes | Yes | Yes | Yes | Yes | Yes | Yes | 7/8 |
| Mackenzie et al. [25] | Yes | Yes | Yes | Yes | Yes | Yes | Yes | No | 7/8 |
| Bastola et al. [26] | Yes | Yes | Yes | Yes | Yes | Yes | Yes | Yes | 8/8 |
| Hookham et al. [27] | No | Yes | Yes | Yes | Yes | Yes | Yes | Yes | 7/8 |
| Kumar et al. [28] | Yes | Yes | Yes | Yes | Yes | Yes | Yes | Yes | 8/8 |
| Aasfara et al. [29] | Yes | Yes | Yes | Yes | Yes | Yes | Yes | Yes | 8/8 |
| Paybast et al. [30] | Yes | Yes | Yes | Yes | Yes | Yes | No | No | 6/8 |
| Ottaviani et al. [31] | No | Yes | Yes | Yes | No | Yes | Yes | Yes | 6/8 |
| Casas et al. [32] | No | Yes | Yes | Yes | Yes | Yes | Yes | Yes | 7/8 |
| Ribeiro et al. [33] | No | No | Yes | Yes | No | No | No | Yes | 3/8 |
| Hutchin et al. [34] | No | Yes | Yes | Yes | Yes | Yes | Yes | Yes | 7/8 |
| Karimi-Galougahi et al. [35] | No | Yes | Yes | Yes | Yes | No | No | No | 4/8 |
| Oke et al. [36] | No | Yes | Yes | Yes | Yes | Yes | Yes | Yes | 7/8 |
| Derollez et al. [37] | No | Yes | Yes | Yes | Yes | Yes | Yes | Yes | 7/8 |
| Hasibi et al. [38] | No | Yes | Yes | Yes | Yes | Yes | Yes | Yes | 7/8 |
| Zain et al. [39] | No | Yes | Yes | Yes | Yes | Yes | Yes | Yes | 7/8 |
| Taouihar et al. [40] | No | Yes | Yes | Yes | Yes | Yes | Yes | Yes | 7/8 |
| Kerstens et al. [41] | No | Yes | Yes | Yes | Yes | Yes | Yes | Yes | 7/8 |
| Ochoa-Fernández et al. [42] | No | No | Yes | Yes | Yes | Yes | No | Yes | 5/8 |
| Kakumoto et al. [43] | No | Yes | Yes | Yes | Yes | Yes | Yes | Yes | 7/8 |
| Pelea et al. [44] | No | Yes | Yes | Yes | Yes | Yes | Yes | Yes | 7/8 |
| Shinde et al. [45] | No | Yes | Yes | Yes | Yes | Yes | Yes | Yes | 7/8 |
| Al-Mashdali et al. [46] | No | Yes | Yes | Yes | Yes | Yes | Yes | Yes | 7/8 |
| Judge et al. [47] | No | No | Yes | Yes | No | No | No | Yes | 3/8 |
| Tran et al. [48] | No | Yes | Yes | Yes | Yes | Yes | Yes | Yes | 7/8 |
| Silveira et al. [49] | Yes | Yes | Yes | Yes | Yes | Yes | Yes | Yes | 8/8 |
| Liberatore et al. [50] | Yes | Yes | Yes | Yes | Yes | Yes | Yes | Yes | 8/8 |

Joanna Brigg’s Institute Critical Appraisal Checklist for Case Series

| Author | Q1 | Q2 | Q3 | Q4 | Q5 | Q6 | Q7 | Q8 | Q9 | Q10 | Total |
| --- | --- | --- | --- | --- | --- | --- | --- | --- | --- | --- | --- |
| Lima et al. [51] | Yes | Yes | Yes | No | No | No | Yes | Yes | No | NA | 5/10 |
| Manganotti et al. [52] | Yes | Yes | Yes | Yes | Yes | Yes | Yes | Yes | Yes | NA | 9/10 |
| Eglimez et al. [53] | Yes | Yes | Yes | Yes | Yes | No | Yes | Yes | Yes | NA | 8/10 |
| Correa et al. [54] | Yes | Yes | No | No | No | No | No | Yes | No | NA | 3/10 |
| Neo et al. [55] | Yes | Yes | Yes | Yes | Yes | No | Yes | Yes | No | NA | 7/10 |
| Khedr et al. [56] | No | Yes | Yes | Yes | Yes | No | Yes | Yes | No | NA | 6/10 |
| Gonzales-Castro et al. [57] | No | No | Yes | No | No | Yes | Yes | Yes | No | NA | 4/10 |
| Bigaut et al. [58] | Yes | Yes | Yes | Yes | No | Yes | Yes | Yes | No | NA | 7/10 |
| Abolmaali et al. [59] | No | No | Yes | No | No | Yes | Yes | Yes | No | NA | 4/10 |
| Kaplan et al. [60] | No | No | Yes | No | No | Yes | Yes | No | No | NA | 3/10 |
